# Essential newborn care practice at four primary health facilities in conflict affected areas of Bossaso, Somalia: a cross-sectional study

**DOI:** 10.1186/s13031-019-0202-4

**Published:** 2019-06-13

**Authors:** Ribka Amsalu, Catherine N. Morris, Kingsley Chukwumalu, Michelle Hynes, Shehryar Janjua, Alexia Couture, Aimee Summers, Amy Cannon, Erin N. Hulland, Sabine Baunach

**Affiliations:** 1 Emergency Health, Department of Global Health, Save the Children, 2275 Sutter Street, San Francisco, CA 94115 USA; 2 Department of Global Health, Save the Children, 899 North Capitol Street NW, Suite 900, Washington, DC 20002 USA; 3 Save the Children in Somalia, Lavington, Nairobi, Kenya; 4 Center for Global Health, US Centers for Disease Control and Prevention, 1600 Clifton Rd., Atlanta, GA 30329-4027 USA; 5 Save the Children in Somalia, Lavington, Nairobi, Kenya; 6 Department of Global Health, Save the Children, 899 North Capitol Street NW, Suite 900, Washington, DC 20002 USA

**Keywords:** Newborn care, Conflict settings, Childbirth satisfaction, Somalia

## Abstract

**Background:**

Newborn mortality is increasingly concentrated in contexts of conflict and political instability. However, there are limited guidelines and data on the availability and quality of newborn care in conflict settings. In 2016, an interagency collaboration developed the *Newborn Health in Humanitarian Settings Field Guide- Interim version (Field Guide)*. In this study, we sought to understand the baseline availability and quality of essential newborn care in Bossaso, Somalia as part of an investigation to determine the feasibility and effectiveness of the *Field Guide* in improving newborn care in humanitarian settings.

**Methods:**

A cross-sectional study was conducted at four purposely selected health facilities serving internally displaced persons affected by conflict in Bossaso. Essential newborn care practice and patient experience with childbirth care received at the facilities were assessed via observation of clinical practice during childbirth and the immediate postnatal period, and through postnatal interviews of mothers. Descriptive statistics and logistic regression were employed to summarize and examine variation by health facility.

**Results:**

Of the 332 pregnant women approached, 253 (76.2%) consented and were enrolled. 97.2% (95% CI: 94.4, 98.9) had livebirths and 2.8% (95% CI: 1.1, 5.6) had stillbirths. The early newborn mortality was 1.7% (95% CI: 0.3, 4.8). Nearly all [95.7%, (95% CI: 92.4, 97.8)] births were attended by skilled health worker. Similarly, 98.0% (95% CI: 95.3, 99.3) of newborns received immediate drying, and 99.2% (95% CI: 97.1, 99.9) had delayed bathing. Few [8.6%, (95% CI: 5.4, 12.9)] received immediate skin-to-skin contact and the practice varied significantly by facility (*p* < 0.001). One-third of newborns [30.1%, (95% CI: 24.4, 36.2)] received early initiation of breastfeeding and there was significant variation by facility (*p* < 0.001). While almost all [99.2%, (95% CI: 97.2, 100)] service providers wore gloves while attending births, handwashing was not as common [20.2%, (95% CI: 15.4, 25.6)] and varied by facility (*p* < 0.001). Nearly all [92%, (95% CI: 86.9, 95.5)] mothers were either very happy or happy with the childbirth care received at the facility.

**Conclusion:**

Essential newborn care interventions were not universally available. Quality of care varied by health facility and type of intervention. Training and supervision using the *Field Guide* could improve newborn outcomes.

## Background

The term ‘humanitarian crisis’ refers to a singular event or a series of events, such as armed conflict or natural disasters that threaten the health, safety or wellbeing of a population [1]. Humanitarian crisis can be acute, within 4-6 months following an event, or protracted [2]. Protracted crises are defined as *“those environments in which a significant proportion of the population is acutely vulnerable to death, disease and disruption of livelihoods over a prolonged period of time. The governance of these environments is usually very weak, with the state having a limited capacity to respond to, and mitigate, the threats to the population, or provide adequate levels of protection.”* [3]. The health impact of a humanitarian crises is evidenced in the excess morbidity and mortality associated directly with the crisis and in the destruction of existing health systems [2]. In recent years, humanitarian crises have intensified in terms of complexity and scale, resulting in the largest global numbers of refugees and internally displaced persons (IDPs) seen in decades [4].

Studies have shown that children under 5 years of age (under-5) experience disproportionally elevated mortality in humanitarian crises [2]. Globally, of the ten countries with the worst neonatal mortality rate, five are experiencing humanitarian emergencies: Afghanistan, Somalia, South Sudan, Central African Republic, and Chad [7]. Earlier studies have associated increased child mortality with specific infectious disease outbreaks in the under-5 population [2, 6]. However, with the decline in child mortality due to infectious diseases, the importance of death in the neonatal period (0–28 days of life) has increased and accounts for nearly 47% of under-5 deaths [7, 11]. Additionally, the average annual reduction rate in neonatal mortality between 1990 and 2016 is much lower than that for children aged 1–59 months [7]. Mortality risk during the newborn period is highest at the time of birth and in the first 24 h of life when over one-third of newborn deaths occur; in total, nearly three-quarters of the mortality among newborns occurs within the first 7 days of life [12]. Inherently, armed conflict leads to disruption of health systems, dislocation of a population, loss of skilled health care workers, financial distress, and disruption in social networks and norms [5]. This disruption could lead to increased vulnerability of pregnant women and their newborns. Despite this high burden of newborn mortality in countries affected by humanitarian crises, special considerations of how to implement evidence-based newborn interventions in these areas are lacking [9]. A systematic review of evidence in public health interventions in humanitarian emergencies, by Blanchet and colleagues, revealed an absence of data on newborn health in humanitarian emergencies [10]. The few studies identified in the systematic review by Blanchet and colleagues - on emergency obstetrics and delivery care, did not yield information on newborn health [10].

In a 2012 survey of providers in humanitarian settings, a lack of training and clinical guidelines was reported as a barriers in the provision of newborn care in humanitarian settings [13]. In response to the lack of technical guidelines and standards on newborn care in humanitarian settings, an interagency collaboration developed the *Newborn Health in Humanitarian Settings: Field Guide (Field Guide)* [14]. The evidence-based interventions referenced in the *Field Guide*, including essential newborn care, are proven to be effective in improving newborn survival, are feasible in low- and middle-income countries, and are recommended by the World Health Organization (WHO) [15].

Essential newborn care as defined by the WHO and explained in the *Field Guide*, are care practices and interventions provided to a newborn immediately after birth comprising thermal care, infection prevention, initiation of immediate breastfeeding within an hour after birth, and newborn resuscitation [15]. These interventions are recommended to be available at the primary health care level and can be provided by trained midlevel providers such as nurses and midwives [15].

Somalia, a country experiencing a protracted crisis, was selected as the site to investigate the feasibility and effectiveness of the *Field Guide* in improving quality of care. More than two decades of civil war in Somalia has resulted in the displacement of 2.5 million persons internally and across the border into neighboring countries [18, 19]. Lack of skilled health workers, low coverage of services, and fragile governance characterize the health care system in Somalia. The Federal Ministry of Health (MOH) was reestablished following the appointment of an internationally recognized government in 2012, after more than two decades of civil-war [20]. Limited financing capability of the MOH has meant that most MOH health facilities benefit from financial and/or technical support from nongovernmental organizations (NGOs) and United Nation (UN) agencies. There are three governance structures - Somaliland, Puntland, and South-Central [20]. While Puntland has had modest success in improving the health status of the population and building health systems compared to South-Central, newborn indicators remain poor [20]. The 2011 UNICEF Puntland Multiple Indicator Cluster Survey (MICS) reported that only 5% of infants received exclusive breastfeeding at six-months of life and only 13 % had an institutional delivery [20]. There is a dire lack of data on current practices during childbirth and the neonatal period in protracted crisis settings such as Somalia. Therefore, understanding the baseline information of current practices and services available, as well as the readiness of health facilities to provide newborn care is critical.

The humanitarian emergency directly affects Puntland:, the United Nations Office for the Coordination of Humanitarian Affairs (UN OCHA), reports instances of armed conflict and associated displacement in large parts of the country including Bossaso, Puntland [19]. Bossaso city hosts an estimated 69,000 IDPs [19], who are mostly in non-camp settlements and concentrated in the city where they have access to health care services at the health centers and the regional hospital.

As part of a larger pre and post intervention study aimed at investigating the feasibility and effectiveness of the *Field Guide* in improving newborn-care practices and quality of care at the primary health facility level, we conducted a baseline cross-sectional assessment. In this paper we share the findings of the baseline assessments. To our knowledge, this is the first study to assess essential newborn practices and health facility readiness to provide newborn care in Puntland, Somalia.

## Methods

### Study Sites & Study Population

In Somalia, the health care system has four levels – hospitals, referral health center, primary  health centers, and health posts. Puntland has eight hospitals and 79 health centers [20]. The health centers are the primary providers of reproductive health services including antenatal, delivery, and postnatal care [20]. In Bossaso city, six health centers and one hospital provide maternal and child services to IDPs and to the host community. In consultation with the Ministry of Health, we purposely selected four out of the six primary health facilities serving IDPs based on predefined selection criteria: the health facilities were open 24-h a day / 7 days a week and had an average of at least 40 deliveries per month.

The selected health facilities are staffed by midwives, nurses, auxiliary nurses, and community midwives (Table 1). The total catchment population of all selected health facilities was estimated at 134,735 persons, including IDPs and the host community.

**Table 1:** Characteristics of Primary Health Centers

	Health Facility 1	Health Facility 2	Health Facility 3	Health Facility 4
Catchment population	32,000	34,171	39,564	29,000
Number of women enrolled in the study	55	95	58	45
Operating hours	24/7	24/7	24/7	24/7
Running water 24/7	Yes	Yes	Yes	Yes
Generator or power supply	Yes	No	Yes	Yes
Labor room functional	Yes	Yes	Yes	Yes
Maternity ward	Yes	Yes	Yes	Yes
Newborn area or table for resuscitation	Yes	No	No	Yes
Skilled health worker available 24/7	Yes	No	Yes	Yes
Healthcare workers				
Midwife	4	3	4	3
Registered nurse	3	3	3	6
Auxiliary nurse	6	6	3	7
Community midwife	2	2	3	0
Distance from referral hospital	2 km	2.5 km	3 km	3.4 km

### Eligibility

Women were eligible to participate if they were between 15 and 49 years of age and presented at one of the study facilities during the project period to deliver a child. Women who were immediately referred to a hospital for delivery were excluded. Women who had a stillbirth or early newborn death were excluded from the postnatal home interview out of respect for the family.

### Study design

We collected observation data over an eight-week period between August and October 2016. There were no marked social events, weather events or civil unrest during the study period. A sample of 203 mother-baby pairs was targeted for the longitudinal study to have sufficient power to detect statistically significant differences in outcomes before and after the implementation of the *Field Guide  -  Essential Newborn Care* component. The sample size was allocated across the four facilities using population proportionate to estimated size (PPES) based on the number of deliveries over a six-month period prior to the study period [21].

We collected data with two instruments– an observation checklist and a structured postnatal interview questionnaire. The observation checklist was adapted from previously validated tools [25, 26] based on the WHO’s Managing Complications in Pregnancy and Childbirth Guide [17]; and the postnatal interview childbirth satisfaction questionnaire and Likert scale were adapted from a previously validated Mackey childbirth satisfaction rating scale [22, 23]. The observation checklist had eight sections: maternal history; health facility labor room preparedness; delivery care and immediate newborn assessment; newborn thermal care; readiness for newborn resuscitation; support and initiation of early breastfeeding; routine care and prophylaxis provided (including pre-discharge education to mother on newborn care); and maternal and newborn outcomes. Observation started at the time of admission of the pregnant woman to the health facility for childbirth until her subsequent discharge from the health facility. The time spent at the health facility for each mother was recorded by trained observers.

### Measures of essential newborn care

Essential newborn care interventions were defined using WHO guidance [15] under 4 categories: 1) thermal care, which consists of immediate drying (drying of newborn with towel at birth), delayed bathing^1^ (newborn did not receive bathing from birth to discharge from the health facility), and skin-to-skin contact (the placement of newborn on mother’s chest after cord cutting with skin-to-skin contact and no barrier between newborn and mother); 2) feeding, which consists of assistance provided to the mother for the initiation of immediate breastfeeding (provider shows mother early signs of attachment to nipple and suckling), and early initiation of breastfeeding (start of breastfeeding within an hour after birth); 3) hygiene, which consists of provider handwashing (provider washes hands with water and soap prior to attending childbirth), provider wearing gloves (provider wears a pair of unused or sterile gloves prior to attending childbirth), clean delivery kit available (delivery kit in sterile wrap, placed on an instrument tray or similar, and ready for use), clean delivery bed available (visibly clean delivery bed ready for pregnant women to deliver on), and dry cord care (nothing placed on umbilical stump and stump is kept uncovered); and 4) readiness for newborn resuscitation, which was measured with the availability of printed partographs to monitor labor, fetoscope/doppler for auscultation, self-inflating resuscitation bag, term mask, clean area in labor room for newborn resuscitation (resuscitation surface), and suction device.

Maternal obstetric history and interventions received during pregnancy were collected through a structured questionnaire, administered to the mother, including antenatal visits, tetanus toxoid vaccination during pregnancy, intermittent preventive treatment of malaria in pregnancy (IPTp), syphilis screening and treatment for urinary tract infection. These evidence-based interventions during pregnancy are associated with reduction in prematurity, low birth weight, and early neonatal infection [16].

### Data collection

We collected data using tablets with KoBo Toolbox software [27]. Sixteen observers were trained on the observation checklist and consent process. We trained the observers using video based illustrations that demonstrate standard practice during labor, delivery, and immediately after birth. All observers were female, from the community, had a health background, and were not employees of the Ministry of Health. The observation tool was piloted for 2 days with pairing of observers to assess inter-observer agreement in observation (exact count agreement was 94.6%)^2^ and to evaluate their performance. To minimize the Hawthorne effect observers were selected from the community, decreasing the possibility of the observer being perceived as an outsider [28], while at the health facility, the observers wore similar uniforms to other health facility staff further creating a non-threatening perception [28, 29]. In addition to the observers, we trained four female interviewers for 4 days on the postnatal interview data collection tool. Postnatal interviews were administered to mothers who consented to be visited at home on days 7–9 after birth discharge from the health center. In the absence of a proper home/residence address, data collectors depended on the phone number that was given by the mother prior to discharge to find the home for postnatal interview. If repeated phone calls were not answered, or if they were informed that the family had moved outside the Bosasso city limits, the mother-baby pair was recorded as lost to follow up.

Data were entered directly on tablets and were uploaded to Kobo Toolbox, and were exported to a Microsoft Excel database (Microsoft Excel 2016) daily. Validation rules were built into the tablet-based survey tool to prompt the observer on missing data or data that required review. Data quality was checked each day by the research coordinator. Any inconsistencies were verified immediately with the observers and were cross-checked with records at the health facility. The data collected did not have personal identifiers and codes used were de-identified prior to data sharing for analysis.

### Statistical analysis

Descriptive analyses were performed and summarized with means and standard deviations for continuous variables and proportions with 95% confidence intervals for categorical variables. Maternal age, parity, number of antenatal visits, tetanus toxoid vaccination, and gestational age were collected as continuous variables and were categorized for analysis. Observation of essential newborn practice and the immediate postnatal period were binary variables. We calculated the proportion and 95% confidence interval of newborns who received interventions. Missing responses were identified and were excluded from the analysis. Logistic regression was used to examine variations in observed practices between health facilities. Statistical significance was defined as *p* < 0.05. The postnatal interview Likert scale score was analyzed by estimating the proportion of response for each category. Recall of educational messages was binary (yes/no) and the proportion with 95% confidence interval was calculated. All analyses were performed using Stata version 14 (StataCorp. STATA Statistical Software: Release 14.0. 2015).

### Ethical approval and informed consent

Approval for the study was sought and obtained from the Puntland Ministry of Health and from the Save the Children ethics review committee, and a non-research determination was approved by The Centers for Disease Control and Prevention (CDC). Women who presented to the health center for delivery care were approached by the study team. Consent information was read to the women in the local language, and verbal consent was sought. Those who consented were included in the study. Personal identifiers collected to facilitate the postnatal follow-up visit were destroyed immediately after completion of the data collection process. Consent was also sought and obtained from health care providers who were working in the four health facilities and who were directly involved in the provision of maternal and newborn care.

## Results

### Obstetric history of study sample

**Fig. 1:**
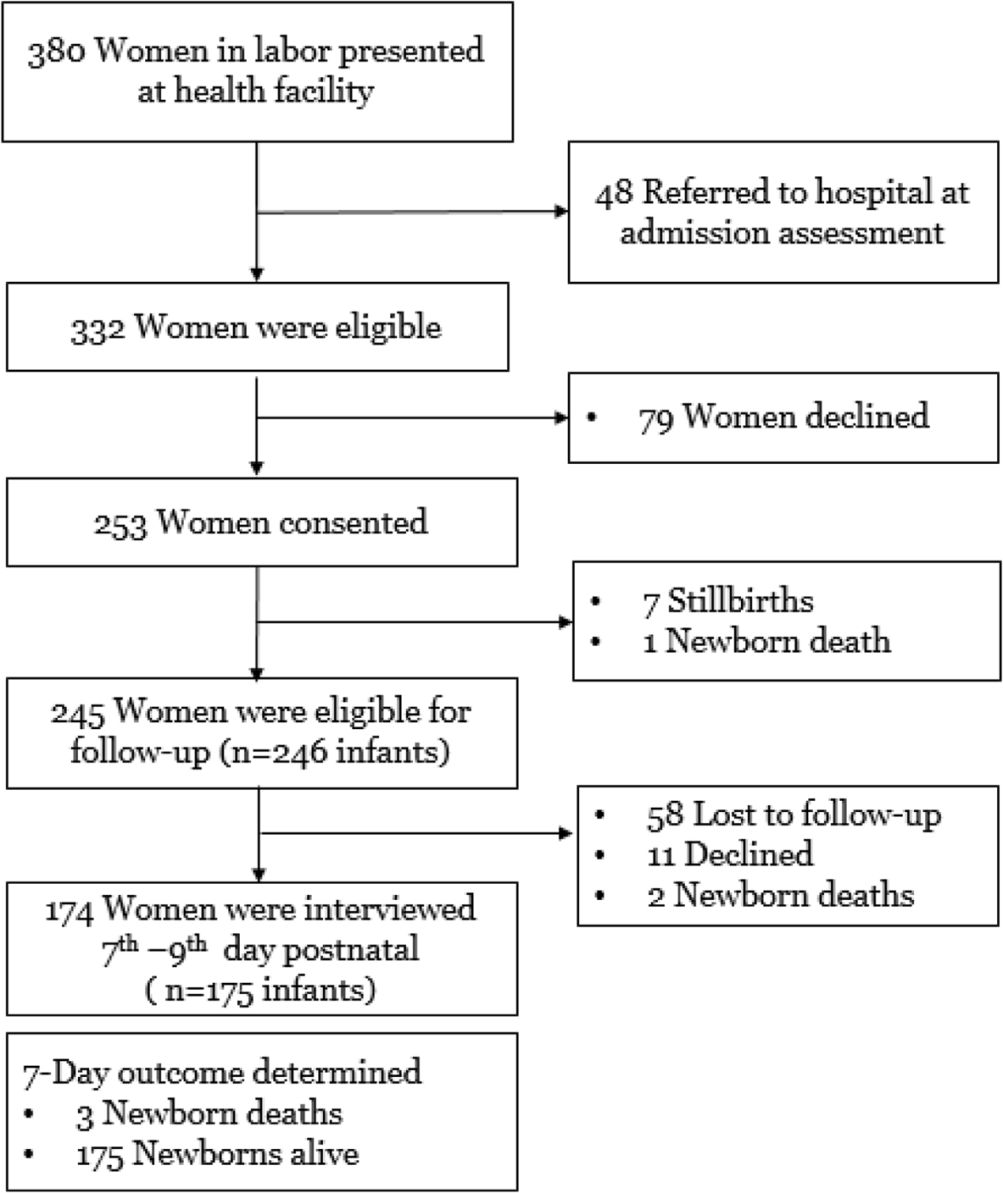
Flowchart of Study Participants

Overall, 380 pregnant women in labor presented at the health facilities during the study period of August–October 2016. Of those women, 48 (12.6%) were referred to a hospital after assessment at arrival and were not approached for consent. Of the 332 women approached for consent, 253 (76.2%) consented and were enrolled in the study. Among the 253 women enrolled, 245 (96.8%) were eligible for home postnatal interview, with 8 participants ineligible due to stillbirth or early neonatal death. Eleven women declined and 58 women were lost to follow-up. Nearly three-quarters (*n* = 174, 71.0%) were successfully interviewed at home 7 to 9 days after birth discharge (Fig. 1).

The median maternal age was 25 years (Interquartile range (IQR): 21–30), and less than one-tenth [9% (95% CI: 5.9, 13.3)] of pregnant women were under the age of 19 years. One-fifth [19.8% (95% CI: 15.0, 25.2)] were primigravida. The majority [87.4%, (95% CI: 82.7, 91.3)] of women had prior contact with the health care system with at least one antenatal visit, and 14.2% (95% CI: 10.1, 19.2) had four or more antenatal visits.^3^ The majority [70.8%, (95% CI: 64.7, 76.3)] of women were diagnosed with a urinary tract infection at an antenatal visit and, of those, 95.5% (95% CI: 91.4, 98.1) received antibiotics. Few [10.3% (95% CI: 6.8, 14.7)] had received intermittent preventive treatment for malaria during pregnancy while fewer still [9.1% (95% CI: 5.9–13.3)] had received screening for syphilis (Table 2).

**Table 2:** Obstetric History & Interventions Received During Pregnancy

	n/N	Proportion (95% CI)
Maternal Age		
15–18 years	23/253	9.1 (5.9–13.3)
19–24 years	78/253	30.8 (25.2–36.9)
≥ 25 years	152/253	60.1 (53.8–66.2)
Gravidity		
Primigravida	50/253	19.8(15.0–25.2)
Two or more pregnancies	203/253	80.2(74.8–85.0)
Antenatal Care (ANC)		
None	31/247	12.6(8.7–17.3)
One to three ANC visits	181/247	73.2 (67.3–78.7)
Four or more ANC visits	35/247	14.2(10.1–19.2)
Interventions during current pregnancy		
Tetanus toxoid vaccination		
None	78/250	31.2 (25.5–37.3)
Received 1 TT doses	98/250	39.2 (33.1–45.6)
Received ≥2 TT doses	74/250	29.6 (24.0–35.7)
Urinary Tract Infections (UTI)		
Had history of symptoms of UTI	179/253	70.8(64.7–76.3)
Received antibiotics for symptoms of UTI	171/179	95.5(91.4–98.1)
Received test for Syphilis	23/253	9.1(5.9–13.3)
Received preventive treatment for Malaria (IPTp)	26/253	10.3(6.8–14.7)

^a^Median maternal age was 25 yrs. with interquartile range of 21 years - 30 years

### Delivery attendants & birth outcomes

The caseload at the four health facilities was similar, with 1–3 deliveries per 24-h period. Most [95.7%, (95%CI: 92.4, 97.8)] deliveries were attended by a skilled provider, usually a qualified midwife or nurse (Table 3). The birth outcomes were mostly singleton spontaneous vaginal deliveries, with one twin birth and no assisted vaginal deliveries. Overall, there were 97.2% (95% CI: 92.4, 98.9) live births, 2.8% (95% CI: 1.1, 5.6) stillbirths, and 1.7% (95% CI: 0.3, 4.8) early newborn deaths (one newborn died prior to discharge at the health facility and two were reported at a postnatal visit to have died in the first week of life). The median gestational age was 38 weeks with 2.4% (95% CI: 0.9, 5.1) of infants born preterm prior to completing 37 weeks of gestation. In terms of newborn complications, 4.5% (95% CI: 2.2, 7.9) of newborns needed resuscitation and all received resuscitation successfully. There were no maternal deaths.

**Table 3:** Birth Outcomes and Newborn Complications

	n/N	Proportion (95% CI)
Birth Attendant		
Skilled health worker ^a^	242/253	95.7(92.4–97.8)
Non-skilled health worker ^b^	11/253	4.3(2.2–7.6)
Birth Outcome		
Live births ^c^	246/253	97.2(94.4–98.9)
Stillbirth	7/253	2.8 (1.1–5.6)
Early newborn death ^d^	3/178	1.7 (0.3–4.8)
Gestational age^e^		
< 37 Weeks	6/252	2.4 (1.9–3.0)
≥ 37 Weeks	246/252	97.6 (94.9–99.1)
Newborn Complications ^f^		
Infection	5/246	2.0 (0.7–4.7)
Fast breathing	4/246	1.6 (0.4–4.1)
Birth Asphyxia	11/246	4.5 (2.3–7.9)
Congenital anomalies	1/246	0.4 (0.0–2.2)

^a^Skilled health workers were Midwives who attended 229/242 or Registered Nurse who attended 13/242 births

^b^Non-skilled health workers were Auxiliary Nurses who attended 9/11 or Community Midwife who attended 2/11 births

^c^One of the deliveries was a twin livebirth

^d^Defined as death within 7 days after birth. 1 death at health facility and 2 deaths at home. 7-day outcome determined among 178 newborns

^e^Missing gestational age of 1 newborn

^f^More than one complication could have been reported by patient. Denominator total livebirths with 1 twin birth

### Essential newborn care by health facility (Table 4)

**Fig. 2:**
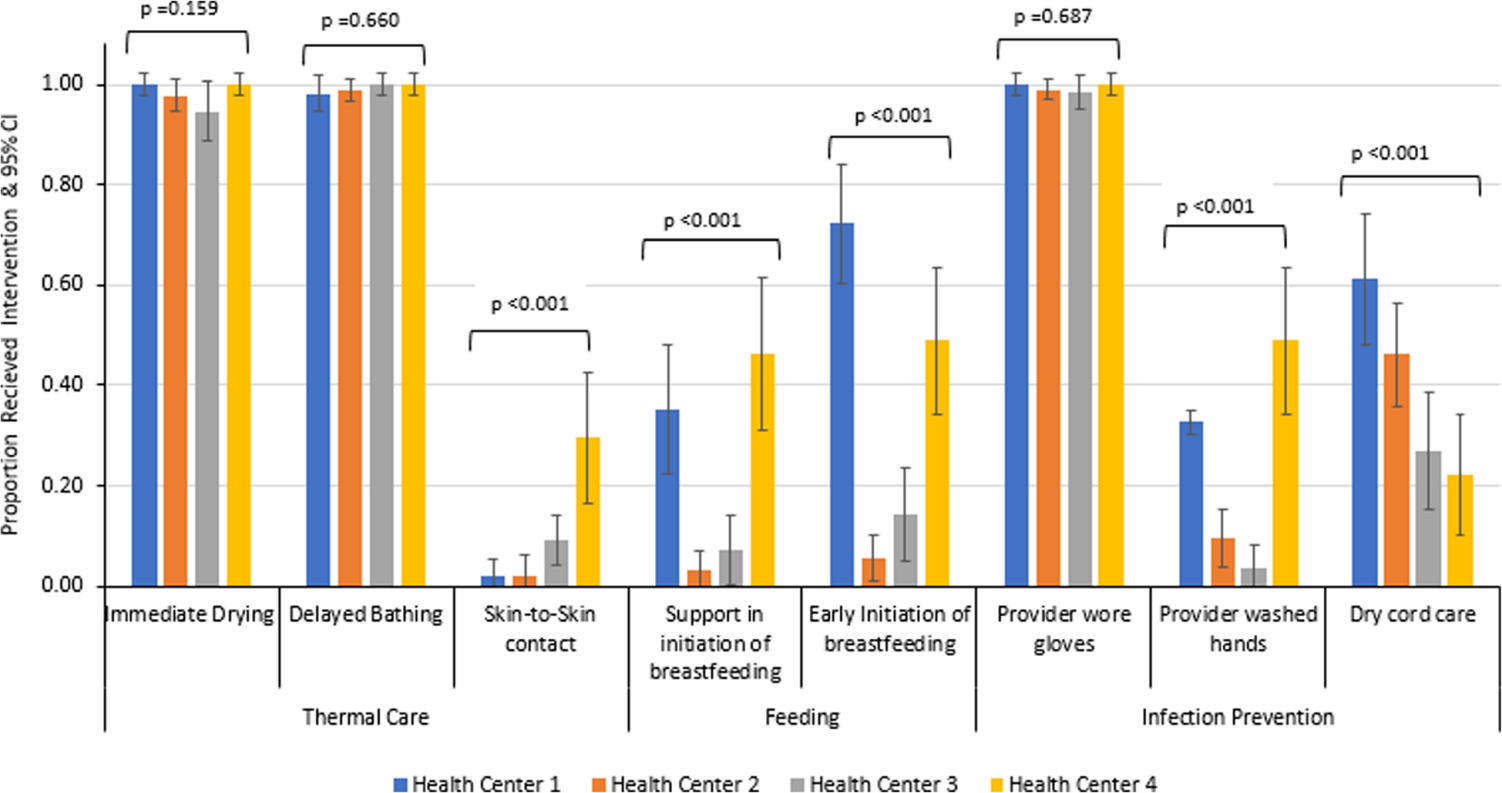
Essential Newborn Care Practice at Four Health Centers in Bossaso, Somalia

The first category of essential newborn care, thermal care, had two of three components - drying immediately after birth and delayed bathing - practiced nearly universally in all the health facilities with an overall proportion of 98.0% (95% CI: 95.3, 99.3) and 99.2% (95% CI: 97.1, 99.9%), respectively. Conversely, the third component of thermal care - skin-to-skin contact of newborns with their mother - was rare, with only 8.6% (95% CI: 5.4, 12.9) observed. There was significant variation between the facilities with a low of 1.9% (95% CI: 0.2, 9.9) in Facility 1 and a high of 29.5% (95% CI: 16.8, 45.2) in Facility 4 (*p* < 0.001) (Fig. 2). There was no statistically significant variation in skin-to-skin contact by parity or gestational age of newborn.

**Table 4:** Observed Essential Newborn Care Practice by Health Facility

	Overall		Health Center 1		Health Center 2		Health Center 3		Health Center 4	
	n/N	Proportion (95% CI)	n/N	Proportion (95% CI)	n/N	Proportion (95% CI)	n/N	Proportion (95%CI)	n/N	Proportion (95% CI)
Thermal Care										
Immediate Drying	241/246	98.0(95.3–99.3)	54/54	100 (97.7–100)	89/91	97.8(92.3–99.7)	53/56	94.6(85.0–98.9)	45/45	100(97.6–100)
Delayed bathing	241/243	99.2(97.1–99.9)	53/54	98.2(90.1–99.9)	90/91	98.9(94–100)	54/54	100(97.7–100)	44/44	100(97.7–100)
Skin-to-skin contact^‡^	21/244	8.6(5.4–12.9)	1/54	1.9 (0.05–9.9)	2/91	2.2(0.27–7.71)	5/55	9.1 (3.0–19.9)	13/44	29.5(16.8–45.2)
Breastfeeding										
Support mother in the initiation of breastfeeding^‡^	45/241	18.7(14.0–24.2)	19/54	35.2(22.7–49.4)	3/90	3.3(0.63–8.6)	4/56	7.1 (2.0, 17.3)	19/41	46.3(27.7–57.8)
Early Initiation of breastfeeding^‡^	74/246	30.1(24.4–36.2)	39/54	72.2(58.4–83.5)	5/91	5.5(1.8–12.4)	8/56	14.3(6.4–26.2)	22/45	48.9(33.7–64.2)
Infection Prevention										
Clean delivery bed^a^	250/253	98.8(96.6–99.8)	54/55	98.2(90.3–99.9)	95/95	100(97.7–100)	57/58	98.3(90.8–99.9)	44/45	97.8(88.2–99.9)
Clean delivery kit^b^	42/252	16.7(12.3–21.9)	2/55	3.6 (0.44–12.5)	9/95	9.5(4.42–17.2)	2/57	3.5(0.42–11.9)	29/45	64.4(48.8–78.1)
Provider wore gloves	251/253	99.2(97.2–99.9)	55/55	100 (99.7–100)	94/95	99.0(94.3–99.9)	57/58	98.3(90.8–100)	45/45	100(97.7–100)
Provider washed hands^‡^	51/253	20.2(15.4–25.6)	18/55	32.7(20.7–46.7)	9/95	9.5(4.42–17.2)	2/58	3.50.42–11.9)	22/45	48.9(33.7–64.2)
Dry cord care^‡^	100/246	40.7(34.5–47.1)	33/54	61.1 (46.9–74.1)	42/91	46.2(35.6–56.9)	15/56	26.8(15.8–40.3)	10/45	22.2(11.2–37.1)
Intrapartum Care & Newborn Resuscitation										
Printed partograph ready	9/253	3.6(1.6–6.6)	1/55	1.8(0.04–9.7)	3/95	3.2(0.66–9.0)	0/58	0%	5/45	11.1 (3.7–24.1)
Fetoscope/doppler ready	68/253	26.9(21.5–32.8)	40/55	72.7(59.0–83.9)	2/95	2.1(0.26–7.4)	14/58	24.1(13.9–37.2)	12/45	26.7 (14.6–41.9)
Resuscitation surface ready	23/246	9.4(6.0–13.7)	0/55	0%	3/91	3.3 (0.69–9.3)	15/56	26.8(15.8–40.3)	5/45	11.1 (3.7–24.1)
Resuscitation bag ready	37/246	15.0(10.8–20.1)	18/54	33.3(21.1–47.5)	8/91	8.8(3.9–16.6)	1/56	1.8(0.05–9.6)	10/45	22.2 (11.2–37.1)
Term mask ready	43/246	17.5(12.9–22.8)	19/54	35.2(22.7–49.4)	10/91	11(5.4–19.3)	1/56	1.8(0.04–9.6)	13/45	28.9(16.4–44.3)
Suction device ready	23/246	9.4(6.0–13.7)	3/54	5.6 (1.2–15.3)	11/91	12.1(6.2–20.6)	2/56	3.6(0.43–12.3	7/45	15.6 (6.5–29.5)

^‡^Statistically significant variation by health facility *p*-value < 0.05

^a^The observers marked on the cleanliness of the delivery bed and if the delivery bed was cleaned prior to the arrival of the pregnant woman

^b^The observers marked yes/no to whether Instruments for birth attendance (delivery kit) are placed in the delivery tray in sterilization wrap in the labor room

The second category of essential newborn care, breastfeeding, had 18.7% (95% CI: 14.0, 24.2) of mothers receiving assistance by a health care worker in the initiation of immediate breastfeeding and 30.1% (95% CI: 24.4, 36.2) of mothers initiating early breastfeeding within an hour after birth. Assistance by a health care worker in the initiation of breastfeeding and early initiation of breastfeeding both varied significantly by health facility (*p* < 0.001). There was no statistically significant difference in the initiation of early breastfeeding or support to breastfeeding by parity.

For the third category of essential newborn care, hygienic practice, nearly all [99.2% (95% CI: 97.2, 99.9)] health workers wore clean or sterile gloves to attend childbirth. However, hand washing was only practiced by 20.2% (95%CI: 15.4, 25.6) of health workers prior to attending childbirth, and dry cord care was practiced for 40.7% (95% CI: 34.5, 47.1) of newborns. Hand washing by the health worker and dry cord care practice both varied significantly by health facility (*p* < 0.001), while sterile glove use was not significantly different between facilities.

Readiness for newborn resuscitation, the fourth category of newborn care, was lacking; printed partograph forms were available in 3.6% (95% CI: 1.6, 6.6) of deliveries, fetoscope or doppler for fetal heartbeat auscultation was available in the labor room during 26.9% (95% CI: 21.5, 32.8) of deliveries, and there was a self-inflating resuscitation bag and term mask in the labor room in 15.0% (95% CI: 10.8, 20.1%) and 17.5%(95% CI: 12.9, 22.8) of deliveries respectively.

### Observed postnatal care at health facility

The observed median length of stay at the health facility after childbirth was 4 h and 37 min (IQR: 2 h and 25 min to 7 h and 8 min). In routine care for newborns, 42.1% (95% CI: 35.9, 48.6) received tetracycline eye ointment while only 4.1% (95% CI: 2.0, 7.4) received vitamin K (Table 5). Health education and information provided to mothers prior to discharge was poor: 11.5% (95% CI: 7.8, 16.2) of mothers received information on hygiene, 10.3% (95% CI: 6.8, 14.8) received information on dry cord care, 9.1% (95% CI: 5.8, 13.4) on danger signs, 3.7% (95% CI: 1.7, 6.9) on skin-to-skin care, and 16.5% (95% CI: 12.0, 21.7) on the importance of breastfeeding.

**Table 5:** Observed Postnatal Care Prior to Birth Discharge

	n/N	Proportion (95%CI)
Length of Stay at Health Facility After Childbirth^a^		
≤6 h	161/242	66.5(60.2–72.4)
6- 11 h	61/242	25.2(19.9–31.2)
≥12 h	20/242	8.3 (5.1–12.5)
Routine Care Pre-Discharge		
Exam of newborn within 30 min after birth	150/246	61.0(54.6–67.1)
Tetracycline eye ointment	102/242	42.0(35.9–48.6)
Vitamin K injection	10/243	4.1(2.0–7.4)
Education Provided on Newborn care to Mother by Health Care Worker Pre-discharge		
Breastfeeding	40/243	16.5 (12.0–21.7)
Hygiene	28/243	11.5 (7.8–16.2)
Dry cord care	25/243	10.3 (6.8–14.8)
Danger Signs	22/243	9.1 (5.8–13.4)
Skin-to-Skin Contact	9/243	3.7 (1.7–6.9)

^a^Median length of stay at the health facility after childbirth was 4 h and 37 min with inter-quartile range of 2 h and 25 min – 7 h and 8 min

### Postnatal interview on childbirth care experience

Out of the 245 eligible women for postnatal home interviews, 174 (71.0%) were successfully followed up at home 7 to 9 days following discharge from the facility. Of the 174 women who were interviewed, less than half [40.2% (95% CI: 32.9, 47.9)] reported receiving information on newborn care from health staff (Table 6). Of the women who reported receiving information on newborn care, 11.4% (95%CI: 5.1, 21.3) of women recalled having received information on dry cord care as presented in Table 6. Overall, of the 174 women interviewed, 62.6% (95% CI: 55.0, 69.8) reported receiving messages on breastfeeding and 13.2% (95% CI: 8.7, 19.2) on danger signs of newborn illness.

**Table 6:** Childbirth Experience and Maternal Knowledge of Newborn Care Practices

Intervention	n/N	Proportion (95%CI)
Recalled receiving information on newborn care from health care provider	70/174	40.2 (32.9–47.9)
Type of information received		
Keeping baby warm	1/70	1.4 (0.04–7.7)
Vaccination	14/70	20 (11.4–31.3)
Hygiene	5/70	7.1 (2.4–15.9)
Dry cord care	8/70	11.4 (5.1–21.3)
Recall receiving information from health care provider on danger signs of newborn illness^a^	23/174	13.2 (8.7–19.2)
Recall receiving information from health care provider on breastfeeding	109/174	62.6 (55.0–69.8)
Childbirth Care Satisfaction		
Overall satisfaction with services		
Somewhat or very satisfied	160/174	92.0 (86.9–95.5)
Would return to this facility again	170/174	97.7 (94.2–99.4)
The health worker provided your baby with the best care possible	151/174	86.8 (80.8–91.4)

^a^Danger signs of the newborn illness: not feeding well, reduced activity, lethargic, fast breathing, fits/convulsions, fever, feels cold

**Fig. 3:**
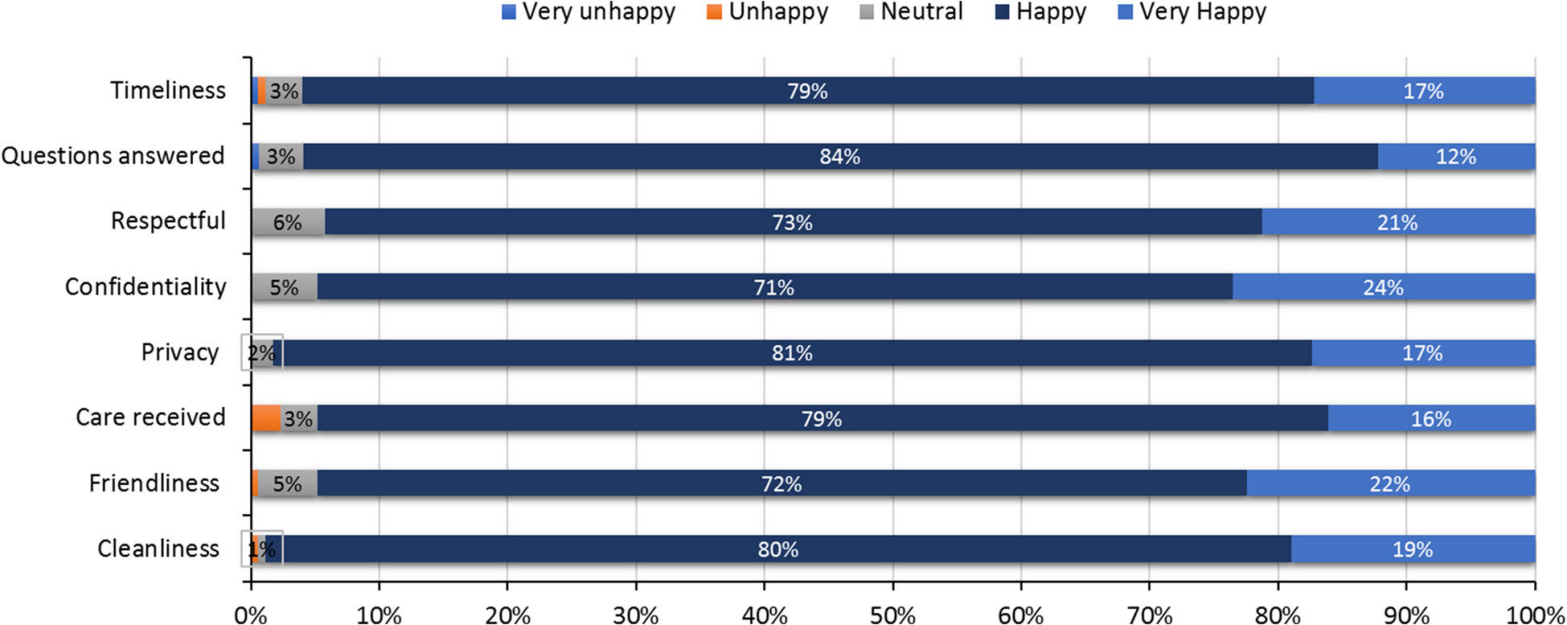
Postnatal Interview of Mothers on Satisfaction with Childbirth Services Received at the Health Facility (*n* = 174)

Across the four health facilities, the majority of women were either happy or very happy in the cleanliness of the clinic [98.9%, (95% CI: 95.9, 99.9)], privacy during service provision [98.3%, (95% CI: 95.0, 99.6)], confidentiality [94.8%, (95% CI: 90.4, 97.6)], friendliness of staff [94.8%, (95% CI: 90.4,97.6)], care received [94.8%, (95% CI: 90.4, 97.6)], response to questions asked [95.9%, (95% CI: 91.9, 98.4)], and timeliness of care provided (96.0%, (95% CI: 91.9, 98.4)] (Fig. 3). Of the five women who responded unhappy or very unhappy, 2 were unhappy or very unhappy in more than one domain and 4 were unhappy with the care provided. Regarding, where the mothers who responded unhappy or very unhappy received care, they were distributed in the three health facilities: 2 mothers delivered in health facility 1; 2 delivered in health facility 2; and 1 delivered in health facility 3.

Overall, 92.0% (95% CI: 86.9, 95.5) of women reported that they were satisfied with the childbirth care provided at the health centers, 97.7% (95% CI: 94.2, 99.4) reported that they would return back to the health facility for future health care needs, and 86.8% (95% CI: 80.8, 91.4) responded that the health care worker provided their baby with the best care available as presented in Table 6. We compared the mother-baby pairs that were visited/followed up postnatally with those that were lost to follow up;, there was no statistically significant difference in place of delivery, maternal age, or birth attendant between the two groups.

## Discussion

Our study, conducted in a protracted conflict setting of Bossaso, Somalia, found that while the majority of the childbirths were attended by skilled health workers (midwives and nurses), the observed quality of care varied. The components of essential newborn care: thermal care, infection prevention, early initiation of breastfeeding, and readiness to provide newborn resuscitation, were not universally provided or available.

Access to health care and a skilled health provider is always a concern in humanitarian emergencies. In our study, the health centers selected were operational 24 h a day/7 day a week and had a skilled health care worker (nurse or midwife) available during all operating hours (Table 1). The percent of women who received at least one ANC visit was high (88%) and was consistent with past studies where IDPs or refugees are concentrated in urban areas where geographic access to a health facility is not an impediment and there are no user fees at the point of care [5, 8]. However, the quality of care found in our study was not optimal. While contact with the health facility as seen with at least one ANC visit was high (88%), the interventions received during pregnancy, such as, screening for syphilis (9%), were very low.

Although the two thermal care components of immediate drying of newborn and delayed bathing were nearly universal for all newborns, skin-to-skin contact was not often practiced. This finding is consistent with studies from sub-Saharan Africa where immediate drying is commonly practiced by birth attendants, but not skin-to-skin contact [25]. Observation studies in Ethiopia, Rwanda, Tanzania, Kenya, and Madagascar also reported less than 60% of women practicing immediate skin-to-skin contact [25]. Similarly, a study in South Sudan, a protracted crises situation, had lower proportions of women practicing skin-to-skin contact at the primary health facility level as compared to the hospital [26]. In our study, skin-to-skin contact was not emphasized on pre-discharge education to mothers and only one mother in the postnatal interview recalled receiving messaging on the importance of skin-to-skin contact. A systematic review by Moore and colleagues [30] showed that skin-to-skin contact when practiced, promoted breastfeeding and stabilization of the healthy term newborn. The benefits of skin-to-skin contact for stability of the cardio-respiratory system and thermal control among low-birth-weight (LBW) and preterm infants have been reported by earlier studies [30, 31], in our study, while 2.4% of births were preterm, there was no statistically significant association between gestational age and skin-to-skin contact.

Overall, less than half (40.2%) of mothers received educational messages on any aspect of newborn care. Studies have shown that mothers are more likely to breastfeed if breastfeeding is initiated early and mothers receive support on how to breastfeed [31]. Though necessity of breastfeeding was the most prevalent observed message delivered to mothers prior to discharge (16.5%) and the most commonly recalled by mothers in the postnatal interview (62.6%), the support provided to mothers to initiate breastfeeding (18.7%) and the percentage that started immediate breastfeeding (30.1%) was much lower than similar studies in conflict settings in sub-Saharan Africa where early immediate breastfeeding was above 90% [25, 26].

Hygienic practices by health care workers during childbirth are vital to reduce health care-associated infections [33]. Nearly all births were attended by health care workers who wore clean or sterile gloves for examination and for attending births. However, handwashing with soap prior to delivery care was practiced by few health workers. The low prevalence of handwashing by health care workers may be due to low adherence to standard practices or low knowledge by service providers. In high neonatal mortality settings such as Somalia, the WHO recommends the application of chlorhexidine (antiseptic gel or solution) to the umbilical stump for infants born at home and dry cord care at health facility level [24]. Our study was focused on deliveries attended at the health facility level, which found that dry cord care was practiced on 41% of infants. In such settings, where hygienic practices are not adhered to, it may be beneficial to consider the use of chlorhexidine for cord care at the facility level.

Intrapartum care and readiness to manage birth asphyxia was low, which is of concern given the number of stillbirths reported (*n* = 7) and the possibility that some of the stillbirths may be due to intrapartum complications or fetal distress that was not detected or managed early [34, 37]. Studies on stillbirths have suggested that intrapartum-related stillbirths, may be preventable with improved intrapartum care with partograph use and early action to manage complications [16].

Length of stay following childbirth in health facilities is recognized as a critical time to educate mothers on newborn care and to identify and manage complications that may arise in the first 24 h [35]. In our study, the median length of stay following childbirth (4 h and 37 min) was shorter than previous studies in developing countries where length of stay ranged from 1.3 days to 6.2 days for vaginal deliveries [35]. The lack of space in the labor room and maternity waiting area could have contributed to the short length of stay. This short length of stay presents a challenge for health care workers in providing key information to mothers before discharge. In our study, few mothers were observed receiving messages on thermal care, cord care, feeding or danger signs and this low level of health messaging was also reflected in the home interviews. This is a missed opportunity, especially in contexts like Bossaso, where mothers may not return for postnatal check-ups and have few additional encounters with the healthcare system. The short length of stay after childbirth and the providers’ level of knowledge and training could have contributed to the low proportion of women that received educational messages.

Most of the mothers interviewed as part of this study reported being happy or very happy with the services they received at the health center. This finding was consistent across the four health facilities and across the eight domains (cleanliness, friendliness, respect, confidentiality, care provided, timeliness, received answer to questions, and privacy). This positive reporting from mothers is encouraging and is higher than in similar studies in sub-Saharan Africa [36]. However, among the five women who responded unhappy or very unhappy, two were unhappy across multiple domains and four out of five were unhappy with care provided. Future studies should follow this qualitative finding with in-depth interviews.

In situations where intrapartum care does not include early recognition of complications by using partographs and timely management of obstetric complications, it is difficult to distinguish deaths due to intrapartum complications from other causes [34]. Therefore, examining both stillbirths and early neonatal deaths is important in evaluating the effectiveness of interventions. Earlier studies on effectiveness of the WHO essential newborn training package have shown statistically significant reductions of early newborn deaths after service providers received the essential newborn training [32, 37]. Training of the midwives and nurses in Bossaso on essential newborn care is crucial to improve quality of care and to decrease newborn mortality.

Our study found significant variation by health facility in the practice of skin-to-skin contact, support in the initiation of breastfeeding, initiation of immediate breastfeeding, handwashing, and dry cord care. Further study is needed to understand what factors contributed to these variations, as the data shows the percent of births attended by a skilled health worker was similar in all four health facilities. Identification of the specific reasons behind the variations will help target support given to facilities and health care workers in order to improve the consistency and quality of newborn care provision in these contexts. Essential newborn interventions are evidence-based lifesaving interventions and it is critical that these interventions are available at quality levels to improve newborn survival in conflict settings.

### Strengths and limitations

This study focused on the provision and quality of essential newborn care provided during labor, delivery and the immediate postnatal period. The findings provide critical baseline information on coverage and gaps of evidence-based newborn care interventions in humanitarian contexts. Using observation of practice rather than clinical records provides greater data accuracy. Observing the delivery of information to mothers by health care providers and asking mothers about what information they recalled receiving approximately 1-week postnatal gives insight on whether key messages are delivered effectively.

The study had several limitations. A high percentage (28%) of mothers were lost to follow-up or refused postnatal interview at home. These women may have had different experiences of satisfaction, maternal knowledge, and newborn complications in the first week of life than those reported by the interviewed women. It is possible that the childbirth satisfaction results underestimated dissatisfaction, as women may have responded positively due to social desirability bias. Additionally, women who were excluded from the interviews because of an adverse birth outcome (stillbirth or early neonatal death) may have had different levels of satisfaction in care. Another limitation is that the presence of observers may have altered the behavior and practice of service providers [38]. The service providers were aware that their practice was being observed for research purposes and may have performed differently than if they were not observed. However, this effect could have been minimized by having observers from Bossaso who were not affiliated with the Ministry of Health and who were on a similar professional level as the observed service providers.

Finally, pregnant women with early signs of complications were referred to the hospital and were not enrolled in this study. It is possible that the findings of the study are an underestimation of maternal and newborn complications and adverse outcomes. In addition, as the health facilities were purposely selected for this study, our findings have limited generalizability.

## Conclusion

In conclusion, this study provides baseline information on essential newborn practice in selected health facilities in Bossaso, Somalia, a protracted conflict situation. Essential newborn care was not universally practiced and there was variation in practice by health facility. The implementation of the *Field Guide* [14] has the potential to improve newborn practice through the training of service providers and the delivery of newborn care education to mothers.

## Abbreviations

CDC: U.S. Centers for Disease Control and Prevention
IDPs: Internally Displaced Persons
WHO: World Health Organization

## Footnotes

1 Delayed bathing, the WHO recommends that bathing should be delayed for at least 6 h from birth. We were unable to ascertain the 6 h delay as some mothers were discharged from the health facility early. This indicator was modified to measure delayed bathing up to time of discharge from health facility.

2 Exact count agreement divided by total count. 3600/3807

3 WHO recommendations on antenatal care for positive pregnancy experience: WHO 2016. Was released at the time of the study. The revised antenatal care model recommends a minimum of eight contacts/visits during pregnancy while the guidance prior to 2016 was four contacts/visits.
